# Efficacy and Safety of Direct Oral Anticoagulants Compared to Warfarin in Prevention of Thromboembolic Events Among Elderly Patients with Atrial Fibrillation

**DOI:** 10.7759/cureus.836

**Published:** 2016-10-18

**Authors:** Shilpa D Kailas, Sirisha Reddy Thambuluru

**Affiliations:** 1 University of Central Florida College of Medicine

**Keywords:** new oral anticoagulants, atrial fibrillation, novel anticoagulants, dabigatran, warfarin, eldery with atrial fibrillation, direct oral anticoagulants

## Abstract

Direct oral anticoagulants (DOACs), previously also known as novel oral anticoagulants (NOACs), have increased the therapeutic options for stroke prevention in atrial fibrillation (AF). Previous studies comparing their relative efficacy and safety do not address age-related differences, such as comorbidities and physical and social boundaries. This review aimed to summarize and compare the clinical and safety outcomes of DOACs and warfarin for stroke prevention in AF in the elderly population (≥ 65 years). We searched PubMed for randomized controlled trials and meta-analyses that compared DOACs and warfarin in elderly patients with AF. Stroke and systemic embolism (SSE) and major bleeding (MB) were primary outcomes. Secondary outcomes included ischemic stroke, all-cause mortality, intracranial bleeding, and gastrointestinal bleeding. Of 66 studies identified, one randomized control trial (RCT) and one meta-analysis were included. DOACs were at least as effective at reducing the risk of SSE as warfarin. DOACs demonstrated a minimal benefit for ischemic stroke (dabigatran, 110 mg, relative risk (RR) 1.08; edoxaban, 60 mg, RR 1.00; and apixaban, 5 mg, RR 0.99). DOACs associated with decreased risk of MB relative to warfarin include dabigatran, 110 mg; apixaban, 5 mg; and edoxaban, 60 mg (RR 0.80, 0.70, and 0.80, respectively), while dabigatran, 150 mg, and rivaroxaban, 20 mg, increased risk (RR 0.79 - 0.83, respectively). Dabigatran, 110 mg and 150 mg doses, and edoxaban increased the risk of gastrointestinal bleeding (RR 1.04, 1.12, and 1.23, respectively). Lower rates of SSE and intracranial bleeding were seen with DOACs compared to warfarin. Dabigatran, 150 mg, and rivaroxaban, 20 mg, were associated with higher MB in older elderly compared to warfarin. DOACs may be attractive alternatives to warfarin, but further studies are needed to make clinical recommendations.

## Introduction and background

Atrial fibrillation (AF) is the most common type of cardiac arrhythmia and is caused by disorganized electrical signals causing irregular contractions of the atria [1]. AF affects more than 2.2 million people in the United States and is expected to double by 2050 [2]. The prevalence of AF increases with age, with a prevalence of 0.1% in the population younger than 55 years, 3.3% of the population 60 years or older, and 10% of the population 80 years or older [3]. A significant complication of AF is stroke; AF increases the risk of stroke by five-fold compared to those without AF [4]. The risk of stroke increases with age as can be seen through the CHA_2_DS2-VAS_c _score, where one point is given to patients 65-75 years old and two points to those older than 75 years. An increase in the number of points indicates a higher risk for stroke. Elderly patients with AF have an overall higher risk of mortality and morbidity due to thromboembolic events, making therapy to prevent stroke critically important [5].

Warfarin, a Vitamin K antagonist anticoagulant, has been the standard medication to decrease thromboembolic events, such as ischemic stroke in AF patients. Although warfarin reduces the risk of stroke by an average of 52% in elderly patients, it is often underprescribed in this population [2, 6]. One factor causing physicians to underprescribe it in the elderly is their increased risk of falls and bleeding [2]. Other important factors that limit compliance with or a prescription for warfarin include regular monitoring of international normalized ratio (INR), variable dosing, food-drug interactions, and drug-drug interactions. These factors may be even more important in the elderly who may be taking multiple medications that may interact with warfarin. Furthermore, physical or mental impairment may make it difficult for them to keep up with INR monitoring sessions [2]. These limitations to warfarin use can leave many elderly AF patients without coverage; this is an important problem as this is the population at high risk for life-threatening thromboembolic events. Recently, direct oral anticoagulants (DOACs), such as rivaroxaban, apixaban, and dabigatran, have been shown to be effective in decreasing stroke risk, with fewer drug interactions, food interactions, easier dosages, and no need for monitoring [7]. These medications can be good alternatives to warfarin, thus, providing a way to get more elderly patients with AF covered and prevent the morbidity and mortality associated with stroke or embolism. Many studies have already looked at the efficacy of DOACs compared to warfarin in patients with AF, but there are limited studies that look solely at the elderly population. The elderly tend to have more physical and social restrictions, as well as more medical comorbid diseases. To extrapolate about the efficacy and safety of DOACs from current studies, which look at all patients with AF, would likely have significant inaccuracies. Hence, this study was conducted to determine the efficacy and safety of direct oral anticoagulants compared to warfarin in the prevention of thromboembolic events only among elderly patients with atrial fibrillation through an evidence-based medicine literature review and summary of a randomized control trial and a meta-analysis study.

## Review

### Search methods

The PubMed online database was used. The keywords “novel oral anticoagulants, direct oral anticoagulants, elderly atrial fibrillation, and stroke” were used to search articles published from January 2000 through November 2015. This resulted in 125 results, most of which looked at solely the direct oral anticoagulants and their effects on the elderly population. Adding “warfarin” as a search term and filtering for only randomized control trials (RCTs) and meta-analysis studies resulted in 92 studies. From this group of studies, we selected a randomized controlled trial and systematic meta-analysis study based on broad study size and patient population, date published, and clinical significance to our target population for the literature review.

### Secondary review of efficacy and safety

Rivaroxaban was evaluated in the double-blinded, randomized control trial, Rivaroxaban Versus Warfarin In Non-valvular Atrial Fibrillation (ROCKET AF), that looked at 14,264 patients with non-valvular AF of various ages randomized to either adjusted-dose warfarin or fixed-dose rivaroxaban and looked at the primary endpoint of stroke or systemic embolism [8]. The current article being reviewed in this study is a secondary analysis of the ROCKET AF study with the purpose of comparing the efficacy (prevention of stroke and systemic embolism) and safety (bleeding) between warfarin and rivaroxaban in patients aged ≥ 75 versus < 75 years [5].

In the participant population ≥ 75 years (n = 6,229), 1,850 patients were randomized to the rivaroxaban group (new therapy) and 1,846 were randomly assigned to the warfarin group. The event rates of the primary endpoint, stroke and systemic embolism, were 2.29% for rivaroxaban versus 2.85% for warfarin per 100 patient-years of follow-up. The hazard ratio for rivaroxaban versus warfarin was 0.08 with 95% Cl (0.63 - 1.02) [5]. The absolute risk reduction and relative risk reduction was 0.0056 and 0.1965, respectively. The number needed to treat was 179 people with rivaroxaban compared to warfarin. For the safety end of point (bleeding events), event rates per 100 patient-years of follow-up were 19. 83% for rivaroxaban and 17.55% for warfarin. The hazard ratio for the safety end point equaled 1.13 with 95% Cl (1.02 - 1.25). The event rates for intracranial bleeding were 0.66% for rivaroxaban and 0.83% for warfarin with a p-value of 0.3531 [5].

Although the quality of the study is not as good as a meta-analysis, there are some significant advantages to this article. For example, the large sample size adds power to the study, the end points were the same as the end points we are looking for in our own study, the secondary analysis was based on a randomized control trial (higher quality study), and the baseline prognostic characteristics were similar to the participant population. There are also some disadvantages to this study. Being a secondary analysis based on another randomized control trial, the original trial may have been designed with different aims. Although the population characteristics differ, the overall aim to compare efficacy and safety of rivaroxaban and warfarin remains the same. Another discrepancy is that our study defines the elderly population as ≥ 65 years of age, but the article in review categorizes elderly as ≥ 75 years; therefore, the data is missing a significant portion of the population in question. Also, the purpose of this secondary analysis differs slightly from our own purpose, in that the secondary analysis’ main aim was to compare the efficacy and safety of rivaroxaban and warfarin between elderly and young participants in the RCT; however, our aim is to solely look at the efficacy and safety of the two therapies in elderly participants.

The ROCKET AF study concluded that anticoagulation therapy with rivaroxaban was as effective as warfarin in reducing stroke and systemic embolism in older patients [5]. The safety events rates for bleeding, as a complication from anticoagulants, was higher in the rivaroxaban group than the warfarin group but with no clinically significant difference. The study concluded that, even though overall bleeding was relatively higher, intracranial bleeding was actually lower in the rivaroxaban group than warfarin but was also not clinically significant in that the P value was greater than 0.05 [5].

### Systemic review of clinical outcomes and safety

Meta-analysis was performed on 25 randomized controlled trials and 24 non-randomized controlled trials from 5,255 total publications. The purpose of this study was to compare the clinical and safety outcomes of oral antithrombotics for stroke prevention in atrial fibrillation in younger (65 - 74 years) and older (≥ 75 years) elderly [9].

Clinical outcomes examined in the systematic meta-analysis include the primary clinical outcome of stroke and systemic embolism (SSE) and secondary outcomes of ischemic stroke and all-cause mortality. The analysis of SSE included 21 RCTs (184,090 patient-years) and non-randomized studies (NRSs) (255,341 patient-years). Direct oral anticoagulants reduced the risk of SSE non-significantly at 22% compared to 18% with warfarin. Comparison between DOACs included dabigatran, 110 mg, dabigatran, 150 mg, rivaroxaban, apixaban, and edoxaban; however, this comparison demonstrated no difference in risk of SSE. Analysis of ischemic stroke included 16 RCTs (179,915 patient-years) and six NRSs (363,322 patient-years) [9]. In this analysis, DOACs exhibited an even smaller benefit for the outcome of ischemic stroke relative to SSE when compared to warfarin. Rate ratios for apixaban and edoxaban were 0.99 (CI 0.56 - 1.17) and 1.00 (CI 0.51 - 1.97), respectively [9]. Dabigatran, 110 mg, conversely demonstrated a nonsignificant association with increased risk of ischemic stroke, with a relative risk (RR) being 1.08 (CI 0.58 - 2.01). The rate ratios for systemic stroke and thromboembolism and the number needed to treat for DOACs compared to warfarin are listed in Table 1 below. Mortality analysis included 20 RCTs (185,023 patient-years) and five NRSs (195,118 patient-years). There was no significant difference in mortality between DOACs and warfarin. Of note, aspirin in the non-reported dose category significantly increased all-cause mortality when compared to warfarin in the overall analyses (RR = 1.69) [9].

**Table 1:** Rate Ratios and Number Needed to Treat for Direct Oral Anticoagulants Compared to Warfarin (a) ^a^ Adapted from Lin, et al., 2015 [9]. Bold values indicate statistical significance (P < 0.05). SSE = Stroke and systemic embolism, CI = Confidence interval, MB = Major bleeding.

Treatment (Vs. Warfarin)	Rate Ratio (CI) for SSE Overall	Rate Ratio (CI) for SSE in RCTs	Rate Ratio (CI) for MB Overall	Rate Ratio (CI) for MB in RCTs	Number Needed to Treat
Dabigatran, 110 mg	0.80 (0.56-1.13)	0.91 (0.67-1.22)	0.96 (0.68-1.34)	0.80 (0.69-0.93)	385
Dabigatran, 150 mg	0.80 (0.62-1.02)	0.66 (0.48-0.89)	0.96 (0.73-1.26)	0.93 (.081-1.06)	104
Rivaroxaban, 20 mg	0.78 (0.55-1.09)	0.74 (0.55-1.00)	0.97 (0.66-1.42)	1.04 (0.91-1.20)	135
Apixaban, 5 mg	0.82 (0.52-1.31)	0.83 (0.65-1.07)	0.78 (0.46-1.30)	0.70 (0.61-0.81)	207
Edoxaban, 60 mg	0.81 (0.46-1.41)	0.85 (0.65-1.12)	0.81 (.045-1.48)	0.80 (0.70-0.91)	234
No Treatment	1.51 (1.10-2.05)		0.88 (0.36-2.16)	0.96 (0.42-2.16)	

Safety outcomes surveyed included the primary outcome of major bleeding as well as gastrointestinal bleeding, intracranial bleeding, and myocardial infarction, secondarily. Major bleeding (MB) was analyzed from 21 RCTs (169,569 patient-years) and eight NRSs (83,375 patient-years). DOACs were found to the reduce the risk of major bleeding by a nonsignificant 3% to 22% relative to warfarin [9]. A study involving only RCT evidence showed dabigatran, 110 mg, apixaban, and edoxaban were associated with a significantly decreased risk of major bleeding compared to warfarin; rate ratios were 0.80 (CI = 0.69 - 0.93), 0.70 (CI = 0.61 - 0.81), and 0.80 (CI = 0.70 - 0.91), respectively. Contrastingly, NRS analysis showed a conflicting trend towards an increased risk of major bleeding with the use of dabigatran, 110 mg. Apixaban and edoxaban were shown to have reduced MB compared with dabigatran, 150 mg, and rivaroxaban (RRs range: 0.67 - 0.87) within subgroups [9]. The rate ratios for MB for DOACs compared to warfarin are summarized in Table 1. When looking at gastrointestinal bleeding in 10 RCTs (154,149 patient-years) and 17 NRSs (517,194 patient-years), dabigatran at both 110 mg and 150 mg doses and edoxaban were associated with an increased outcome (RR 1.04, 1.12, and 1.23, respectively). To assess intracranial bleeding, 12 RCTs (160,812 patient-years) and 14 NRSs (555,037 patient-years) were reviewed. The DOACs had a significantly decreased risk of intracranial bleeding versus warfarin (RRs range: 0.39 - 0.73). Rivaroxaban appeared to have the least benefit of minimizing intracranial bleeds within this group (RR 0.73) [9]. Lastly, the analysis of myocardial infarction (MI) included 15 RCTs (181,221 patient-years) and three NRSs (60,251 patient-years). This analysis showed dabigatran, 110 mg and 150 mg, had an association with greater MI risk than warfarin in RCTs (RR 1.36 and 1.40, respectively). Interestingly, NRS data showed a lower risk of MI with dabigatran use relative to warfarin use [9].

With respect to the population subgroups of younger elderly (≤ 65 years) and older elderly (≥ 75 years), some trends in data were observed upon the cross comparison. NRS data indicated that DOACs (dabigatran, 110 mg and 150 mg, and rivaroxaban) showed a trend toward a smaller risk of ischemic stroke than warfarin with more benefit in the older elderly than the younger elderly (RRs range: 0.85 - 0.85 and 0.76 - 0.81, respectively) [9]. However, this same group of DOACs was more likely to cause major bleeding in the older elderly when compared to warfarin. Dabigatran, 150 mg, was also noted to increase GI bleeding to a greater degree than warfarin in the older but not in the younger elderly group.

This meta-analysis has several advantages. It is a high power study, involving 49 randomized and nonrandomized studies consisting of more than 500,000 patient-years of data. As such, it overcomes the lack of studies directly comparing oral anticoagulants and provides an evidenced-based treatment approach that includes both clinical and safety outcomes. It included real-world assessments of the benefits and harms of DOACs and warfarin, which were generally consistent between RCTs and NRSs and widely observed. Also, as with any study of its type, a broader and more heterogeneous range of patients are examined than any single study alone. Although this systematic review is large scale and comprehensive, it possesses some weaknesses. The use of an open-label design and non-randomized studies may have skewed data points and introduced selection and other biases. NRSs included in the study had low HAS-BLED scores, which indicate that the population was carefully selected and unrepresentative of real-life practice. Thus, it is difficult to generalize safety and efficacy of DOACs from this data when extrapolating for the general population. Furthermore, potential misclassification of outcomes and drug use in the non-randomized data could affect results, although all treatments would be affected equally. To minimize this, upon review, we focused on overall combined RCT and NRS data and RCT data alone. This was done as discrepancies between RCT and NRS data existed. This study includes antiplatelet agents, which are not being examined in our analysis, so its scope is larger. Even with its limitations, this meta-analysis included real-world data and was more inclusive in terms of competing interventions and outcome measures than previous studies of its kind.

This study concluded that lower rates of SSE and intracranial bleeding were seen with DOACs than with warfarin [9]. Two DOACs, dabigatran, 150 mg, and rivaroxaban, were associated with high rates of major bleeding in the older elderly [9]. The older elderly, although at highest risk for major bleeding, also seemed to benefit the most from therapy with DOACs.

### Discussion

Both articles presented similar conclusions: direct oral anticoagulants are just as effective at preventing stroke or systemic embolic events as warfarin. However, DOACs may potentially carry more risk of major bleeding than warfarin, although certain DOACs are associated with less risk of intracranial bleeding. Both articles were valid and reliable in their own right, although the meta-analysis was superior to the secondary analysis as it was classified as Level I evidence, while the secondary analysis of the RCT was Level II [10]. The meta-analysis article analyzed several DOACs (dabigatran, apixaban, apixaban, etc.) and had a wider age range for the elderly sample population (≥ 65 yrs) compared to the secondary analysis, which only looked at rivaroxaban and defined their elderly sample population as ≥ 75 yrs. These differences allow the results of meta-analysis to apply to the larger elderly population, as well as to different DOAC drugs.

Considering the limitations to warfarin, such as frequent INR checks and drug interactions, DOACs, possessing the same efficacy, may seem like attractive alternatives. There are other factors to consider, such as the lack of long-term studies. Warfarin has been in use for a long period of time, and there is a large amount of evidence for its efficacy and knowledge of side effects. These DOACs are still relatively new, and only a few of them are currently FDA approved: dabigatran, apixaban, and rivaroxaban [11]. Also unlike warfarin, many of the DOACs, except for dabigatran, do not have an FDA approved reversal agent to stop active bleeding, which is a crucial limiting factor as major bleeding risk has shown to be increased with DOAC use. New reversal agents for the new oral anticoagulants are in development or awaiting FDA approval. These agents include idarucizumab, andexanet, and PER977 and, thus, make the DOACs attractive alternatives for the future [12]. Yet, recent cost-benefit analysis studies for DOACs vs warfarin are conflicting on which therapy is cost effective. Two studies concluded that warfarin was more cost effective, with one article stating that dabigatran, 150 mg, cost $20,797 more than warfarin adjusted for cost per quality-adjusted-life-year gained [13-14]. Another article states that, in the long-term with statistical analysis of a hypothetical population, medical costs are actually reduced with DOACs [15]. Yet, all of the articles agree that realistic medical costs depend on individual patient characteristics [13-15]. In addition, patient age, in terms of younger versus older elderly, is a consideration for major bleeding risk and potentially devastating consequences with DOACs.

## Conclusions

Taking all this into account, it may not be appropriate to state that DOACs should replace warfarin as the first line but, rather, DOACs are good alternatives to patients who are limited by the disadvantages of warfarin. Elderly patients with non-valvular AF are likely on multiple drugs and physically unable to go to regular INR checks at warfarin clinics. This makes the elderly population prone to difficulties with medication compliance and keeping up the variable warfarin dosages. These factors increase the risk of bleeding in this patient population. Easier dosing, decreasing the risk of intracranial bleeding, decreasing drug interactions, and more reversal agents in the pipeline for approval should make DOACs good alternatives to increase compliance and decrease complications of atrial fibrillation. However, higher level studies, long-term studies, and cost-benefit analysis studies are still needed to make strong clinical recommendations.
